# Experimental and Numerical Thickness Analysis of TRIP Steel under Various Degrees of Deformation in Bulge Test

**DOI:** 10.3390/ma15062299

**Published:** 2022-03-20

**Authors:** Emil Spišák, Janka Majerníková, Ľuboš Kaščák, Peter Mulidrán, Vladimír Rohaľ, Róbert Bidulský

**Affiliations:** 1 Institute of Technology and Materials Engineering, Faculty of Mechanical Engineering, Technical University of Košice, Mäsiarska 74, 04001 Košice, Slovakia; janka.majernikova@tuke.sk (J.M.); lubos.kascak@tuke.sk (L.K.); peter.mulidran@tuke.sk (P.M.); vladimir.rohal@tuke.sk (V.R.); 2 Asian Innovation Hub, Budulov 174, 04501 Moldava nad Bodvou, Slovakia; robert.bidulsky@asihub.org; 3 Bodva Industry and Innovation Cluster, Budulov 174, 04501 Moldava nad Bodvou, Slovakia

**Keywords:** bulge test, numerical simulation, thickness evaluation, TRIP steel, yield criteria

## Abstract

To design a reliable forming process it is necessary to determine the mechanical and formability properties of the processed material, which are used as input parameters for forming simulations. High-strength steel is irreplaceable as a material for producing the deformation zones of current automobiles. This type of steel can be processed by conventional or unconventional forming methods. In the sheet forming process, the material is usually under uniaxial and biaxial stress. The bulge test is utilized for determination of biaxial stress–strain curves, which are often used as input material data for forming simulations. In this work, numerical simulations of bulge tests using TRIP RAK 40/70 steel were performed to study the impact of yield criteria and hardening laws on the accuracy of thickness prediction of the deformed steel sheet. Additionally, the impact of different solvers and integration schemes on the thickness prediction was tested. Furthermore, the impact of various degrees of deformation (various dome heights) on thickness prediction accuracy was evaluated. Numerical results showed a good correlation with experimental data. When the Hill90 yield criterion was used, the software with implicit solver was more accurate in predicting thickness compared to software with explicit integration scheme, in most cases. In addition, the thickness prediction of parts with lower deformation was more accurate compared to parts with greater deformation (higher dome height).

## 1. Introduction

The automotive industry is currently one of the fastest growing industries in the world. Demands to reduce vehicle weight have led to the development and application of high-strength steels in the last three decades. Car body weight reduction requirements reduce fuel consumption and thus reduce CO^2^ emissions. It is for the above-mentioned reasons that steel sheets with higher strength properties, which are characterized by good plasticity, have been widely used in recent years in the production of car bodies [1,2,3,4,5,6].

One such steel that has been developed and used in recent years in the production of car bodies is the plastic-induced steel “TRIP”. These steels have higher strength and have sufficient plasticity in cold forming. During cold plastic deformation of these materials, austenite transforms into deformation-induced martensite. This contributes to the overall strengthening of the pressed sheet [2,5,7]. Sheets made of these steels are characterized by uniform plastic deformation during almost the entire plastic deformation. In contrast to conventional low carbon steels, in the case of TRIP steels local deformation at critical points of the pressings is not so significant [6,7,8].

The uniaxial tension test is used to measure mechanical and elasto-plastic properties of materials, but in real forming processes the biaxial stress state is also present. Thus, it is important to test materials in this type of stress state to accurately predict their deformation behavior in real stamping applications [9,10]. The bulge test is a commonly used test for determining the deformation properties of steel sheets under biaxial stress. The bulge test enables stress and strain determination up to the failure of the sheet metal specimen, unlike the common uniaxial test, which can use only the uniform strain range. Thus, the bulge test is more suitable for description of the plastic properties of sheet metal at large deformations. This is crucial in determining the stress–strain behavior of steel sheets produced in cold-rolled conditions [9,10,11,12,13,14].

The research work of Slota et al. [15] was aimed to determine stress–strain curves of various deep-drawing quality steels under biaxial loading using the hydraulic bulge test. Atkinson [16] studied the stress–strain relationship on the bulge test specimens. In his work, he proposed to use explicit formulations of thickness variations and radius curvature at the pole of the bulge, which change the stress–strain behavior. Spišák et al. [17] conducted research on tin-coated TH415CA steel, which is used in the packaging industry. They analyzed the plastic and mechanical properties of this steel under uniaxial and biaxial loading. Majidi et al. [18] performed experimental and numerical research of the AZ31B magnesium alloy using the bulge test. Their numerical results of the superplastic deformation behavior of magnesium alloy correlated with the results from the experiments.

The mechanical and plastic properties of the materials are necessary input parameters for the finite element analysis (FEA) of forming processes. FEA is an established tool for the calculation and analysis of sheet deformation [13,14,15,16]. The simulation program should be efficient, accurate and easy to use [17,18,19]. Work by Majidi et al. [18] was focused on accurate modelling of the superplastic forming process. They have used the Variable m-value Viscoplastic (VmV) model in ABAQUS software. The proposed model was effective in reproducing superplastic behavior. Di Pietro et al. [19] performed analysis of forming parameters involved in the plastic deformation of stainless-steel tubes using Finite Element Method (FEM). Their results suggest that FEM can be a useful tool in predicting and properly designing industrial deformation processes. The results obtained from sheet metal stamping simulations can be used to adjust the forming conditions (lubrication, blank holder force) as well as the geometry of the forming tool, thus reducing the risk of splits and wrinkles, and minimizing the change in shape and dimensions of the stamping due to springback [20,21,22]. A paper published by Neto et al. [22] was focused on the evaluation of boundary conditions on the prediction of springback and wrinkling in sheet metal forming. They used two types of steel – dual phase and mild-drawing quality steel in the experimental and numerical study. Their numerical results are in good agreement with the experimental ones when the full blank was used in the simulation.

Based on current knowledge, there are four categories of parameter that are vital for accurate prediction in numerical simulation [23,24,25], namely: process, material, geometry, and numerical parameters. The impact of the above-mentioned parameters on forming simulation accuracy is studied and analyzed by several researchers. Neto et al. [22] studied the effect of boundary conditions on the springback and wrinkling prediction of the stamping made of DC06 and dual-phase DP600 steel. They achieved a good correlation of numerical prediction with the experimental results when the simulation used the full blank. Tomáš et al. [26] conducted an FE analysis of stamping a product with the shape of a box, to compare the changes in thickness with experimental results. Hill48 and Hill90 yield criteria, in combination with Krupkowski and Hollomon hardening laws, were used in the simulation. Based on a comparison of the numerical results, they found that the Hill48 yield surface model, in combination with the Krupkowski hardening law, deviated marginally from the experimental results. Habibi et al. [11] performed an experimental and numerical investigation to study failure mechanisms in TRIP steel. They tested and evaluated various damage criteria models which are used for the construction of forming limit diagrams (FLDs). The work of Mulidrán et al. was aimed at the simulation of the springback effect of aluminum-alloy car body parts. Numerical simulations were performed using different theories of plasticity and yield criteria (Barlat89, Barlat2000, Vegter-Lite, Hill90, Hill48 isotropic and Hill48 orthotropic), combined with the Voce hardening law. The geometry comparison was performed in three areas of the sample and a comparison of the predicted results and experimental values was made [27].

The presented work deals with the comparison of bulge test simulation results of RAK 40/70 TRIP steel obtained using two software programs that are often used for prediction and analysis of forming processes. The impact of different yield surface models and hardening models on the simulation accuracy was also studied. The predicted parameters obtained by the numerical simulation and the measured results were compared. Optical and conventional methods were used to measure the thickness of samples. The change in the thickness of the material of the resulting bulge at different degrees of deformation was compared. The novelty of the presented work is the comprehensive analysis of the effect of input material, which combines numerical thickness prediction and the bulge test results using specimens made of TRIP steel under various degrees of deformation.

## 2. Materials and Methods

### 2.1. Material

In the experiments for this work, a 0.75 mm thick steel sheet made of TRIP steel RAK 40/70 Z100MBO (U.S. Steel Košice, Košice, Slovakia) was used. The sheet was hot-dip galvanized. TRIP steels have been used for experimental research because their structure differs significantly from that of low-carbon and low-alloy steels. The structure of these steels consists of ferrite, bainite, martensite and residual austenite. The plastic deformation, therefore, takes place on other principles compared to mild steels. Residual austenite is transformed into martensite in deformation and is found in a polygonal ferrite matrix including bainite, which retains very good ductility [9].

The material’s microstructure affects the process of plastic deformation and the material’s strengthening during plastic deformation. We assume a different mechanism of hardening and plastic deformation than in low-carbon and low-alloy steels. Different materials, therefore, behave differently and have certain specific properties in elastic and plastic deformation [28,29,30,31,32,33].

In order to obtain objective results from Finite Element (FE) simulation of the forming processes, it is necessary to enter as inputs the characteristic properties for a specific type of steel sheet [30]. Therefore, results obtained in other studies and experiments are not applicable to other material types. The chemical composition of the TRIP steel used for the experiments is shown in Table 1. The material’s mechanical and plastic properties were determined by a uniaxial tensile test on standardized specimens.

**Figure 1:**
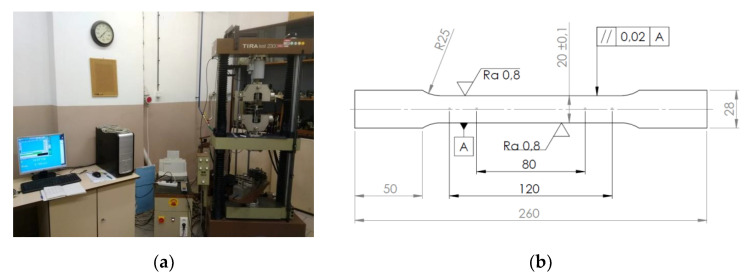
The uniaxial tensile test: Testing machine TIRAtest 2300 (**a**); Specimen dimensions for the test (mm) (**b**).

The measured properties of the material determined by the tensile test are shown in Table 2. To assess the anisotropic properties of the test material, test specimens for the uniaxial tensile test were taken in various orientations (0°, 45° and 90° to the rolling direction). Samples for the uniaxial tensile test were prepared in accordance with the standard STN EN ISO 6892-1: 2020 (Figure 1b). The material properties were determined in accordance with these standards: mechanical properties according to STN EN ISO 6892-1: 2020, normal anisotropy ratio according to STN EN ISO 10113: 2020 and strain-hardening exponent according to STN EN ISO 10275: 2021. Tensile tests were conducted on a VEB TIW TIRAtest 2300 (TIRA Maschinenbau GmbH, Rauenstein, Germany). The outputs from the device (force—elongation—change of sample width) were processed using our software (Figure 1a).

As mentioned above, to obtain the properties of the examined sheet, average values given in Table 2 were determined from five test samples in each of the measured directions (0°, 45° and 90°). The elongation value was measured with a length extensometer (TIRA Maschinenbau GmbH, Rauenstein, Germany), and an extensometer (TIRA Maschinenbau GmbH, Rauenstein, Germany) was used to measure the width of the test specimen. The extensometer manufacturer states an accuracy of ± 0.001 mm for both types of extensometers. To determine the value of the strain-hardening exponent, an elongation of 5% to 15% of the measured length (80 mm) was determined.

The average values and planar anisotropy of the plastic strain ratio and the strain-hardening exponent were calculated with the use of the following equations:

(1) $$r_{m}=\frac{1}{4}\left(r_{0{}^{\circ}}+2\cdot r_{45{}^{\circ}}+r_{90{}^{\circ}}\right),$$

(2) $$\Delta r=\frac{1}{2}\left(r_{0{}^{\circ}}-2\cdot r_{45{}^{\circ}}+r_{90{}^{\circ}}\right),$$

(3) $$n_{m}=\frac{1}{4}\left(n_{0{}^{\circ}}+2\cdot n_{45{}^{\circ}}+n_{90{}^{\circ}}\right),$$

(4) $$\Delta n=\frac{1}{2}\left(n_{0{}^{\circ}}-2\cdot n_{45{}^{\circ}}+n_{90{}^{\circ}}\right).$$

### 2.2. Hydraulic Bulge Test

**Figure 2:**
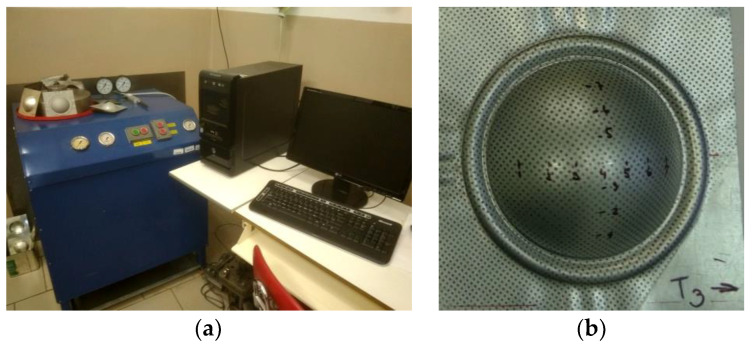
Bulge test device (**a**) and a specimen after the bulge test (**b**).

To compare the properties of the tested material under different stress and strain schemes, a bulge test was used in the experiment. The test was performed on equipment of our design. This device is shown in Figure 2.

The advantage of the designed equipment for the bulge test is the fact that a test specimen is used for the test—sheet metal with dimensions of 130 mm × 130 mm. The average values from this test were determined using 6 test samples for 6 various degrees of deformation (bulge heights). The diameter of the die in which the hemispherical dome is formed is 80 mm. This diameter was chosen because it corresponds to the length of the measured part of the test specimen at uniaxial tension (l_0_ = 80 mm). The test specimens were deformed gradually to a height corresponding to the deformation for determining the strain-hardening exponent by a uniaxial tensile test (5–15%). The samples were labeled sequentially from T8 to T3. Samples with T8 designation were deformed up to 5.2 mm bulge height, samples T7 were deformed up to 9.9 mm, samples T6 were deformed up to 13.5 mm, samples T5 were deformed up to 14.4 mm, samples T4 were deformed up to 18.8 mm and samples T3 were deformed up to 19.3 mm.

### 2.3. Measurement of Sample Thickness Using a Digital Point Micrometer

**Figure 3:**
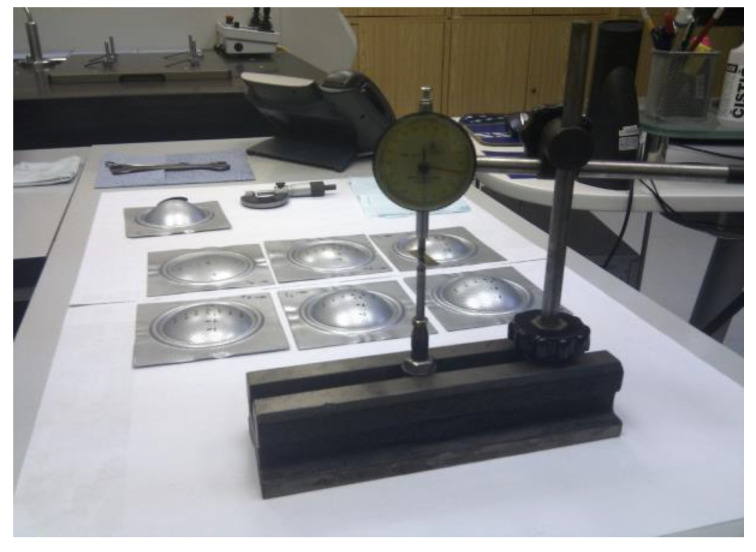
Measuring the thickness of a specimen after the bulge test.

Before the hydraulic bulge test, a net was etched using an electrochemical process on the blanks for the optical measurement. Samples have been measured in 10 mm intervals. The measurement was conducted in 0 to 80 mm distance from the bulge edge in a parallel and perpendicular direction to the direction of rolling (Figure 3). After the bulge test, the thickness of the sample in the marked points was measured using a digital point micrometer Mitutoyo 342-251-30 (Mitutoyo, Sakado, Japan). The thickness measurement in each 10 mm interval was conducted three times and the arithmetic mean of the thickness values was computed. To ensure better access to the measured points, the samples were divided into two parts. This was carried out after optical measurement on the bulge test samples.

### 2.4. Measurement of Sample Thickness Using the Optical Measuring System ARGUS

To measure sheet metal parts using the GOM ARGUS optical measuring system (GOM GmbH, Braunschweig, Germany), it is needed to take images of the sample in various positions and angles. Then, all the acquired high-resolution images and 2D coordinates of all the dots are mathematically derived and recalculated to 3D coordinates using photogrammetry principles, taking into account ray intersections, camera positions, and lens distortion. The main result is a fine 3D point cloud, consisting of a large number of points, which represent the 3D surface of the sheet metal part. By evaluating the relative distance between points, the stress, strain and thickness of the deformed sample can be calculated and evaluated.

**Figure 4:**
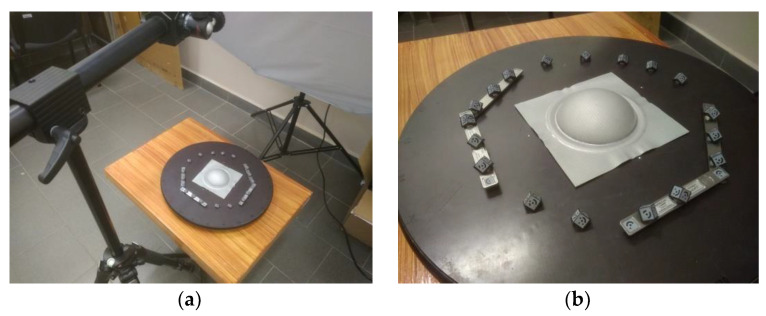
ARGUS measuring system (**a**) and a sample prepared for measurement (**b**).

During bulge testing, the points of the deformation grid on the sample in the deformation areas shifted. The diameter of one grid point was 1 mm and the distance between points was 2 mm. The images of the samples were taken using a digital camera Schneider KREUZNACH CINEGON 1.4/12 (Schneider Optische Werke GmbH, Bad Kreuznach, Germany). Taken images were processed using GOM Inspect software (GOM GmbH, GOM Inspect 2012, Braunschweig, Germany) and the thickness of the bulge test samples was evaluated (Figure 4).

### 2.5. Numerical Simulation of the Bulge Test in FEM Software

The plastic deformation process in the bulge test was simulated using two FEM forming programs that are often used by the automotive sector. The same bulge test input data were used to compare the two selected software systems. Both types of software are based on the finite element method. Two types of software were used to compare the simulation results – one with the implicit solver and triangular elements and the other with the explicit solver and hexagonal elements. In both cases, the deformation process model of the bulge test was created so that the specimen flange was firmly fixed, to prevent the test specimen material from sliding under the flange area. The plastic deformation took place at the expense of the change in the thickness of the material in the die with 80 mm diameter. The accuracy of the numerical simulation was set to fine. The default size of the blank shell element was user-defined and set to 3 mm, and maximum refinement level of the element was set to 3 (minimal size of the element after refinement is 0.375 mm). Radius penetration was set to 0.16 and the number of integration points in the sheet metal thickness was set to 5. The plastic deformation was carried out using the pressure of the hydraulic fluid supplied under the test specimen. In both types of software, the input data on the hardening curves (according to Hollomon and Krupkowski) were used and the yield criteria according to the Hill48 and Hill90 models were set. The numerical simulation aimed to analyze the differences in the predicted thickness of deformed specimens caused by the change of the input hardening laws and plasticity models, which in the simulation represent the tested material - TRIP steel.

#### 2.5.1. Yield Surface

The transition of material from elastic deformation to plastic is most often defined by the yield strength. The yield strength may vary with different stress patterns. The different values will be for uniaxial and biaxial tensile stress of the sheet [34]. From the geometric interpretation of the currently known models of plasticity of the material, it is clear that the critical shear stress at which plastic deformation occurs depends on the magnitude of the applied stresses. For the above reasons, the yield strength value obtained by the uniaxial tensile test is often considered to be an insufficient value of the input to simulation software to ensure that objective results of the tensile process simulation are obtained [35].

**Figure 5:**
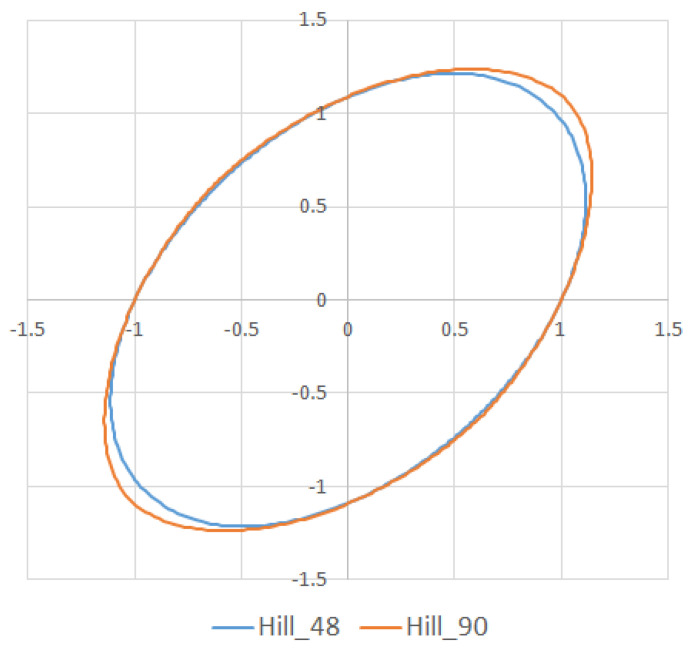
Comparison of Hill48 and Hill90 yield criteria used in FE analysis.

Two yield models were examined in this research. A Hill48 yield surface model and Hill90 yield criteria were used to evaluate the influence of the yield criterion on the simulation accuracy (Figure 5). The anisotropy parameters for the abovementioned yield criteria used in the simulation are specified in Table 3.

The obtained simulation results were analyzed in comparison with the sample thickness values measured with the optical measuring system (ARGUS) and the thickness values measured manually using the digital micrometer.

#### 2.5.2. Hardening Law

The hardening law is necessary to completely define material behavior during deformation. The hardening of the material during cold plastic deformation depends mainly on the structure and microstructure of the formed material. There exist three main types of rules describing material hardening [36]. Isotropic hardening is described by the yield surface expansion when stress is applied, but the yield surface shape remains the same. Kinematic hardening is the second common type. The yield surface remains the same shape and size, but translates in stress space. The third type of hardening behavior is combined hardening – isotropic and kinematic hardening.

In the presented work, two isotropic hardening laws were used in numerical simulations. Isotropic hardening rules are defined as:

- Hollomon
  (5) $$\sigma =K\;\cdot \;\varphi^{n}$$
- Krupkowski
  (6) $$\sigma =K\;\cdot \;{\left(\varphi_{0}+\varphi_{pl}\right)}^{n}$$
  where *σ* represents the true stress, *K* is the strength coefficient, *n* is the strain-hardening exponent, *φ*_0_ is the pre-strain and *φ_pl_* represents the plastic strain. Material model constants used in both hardening rules can be seen in Table 4.

## 3. Results

### 3.1. Experimental Bulge Test Results

**Figure 6:**
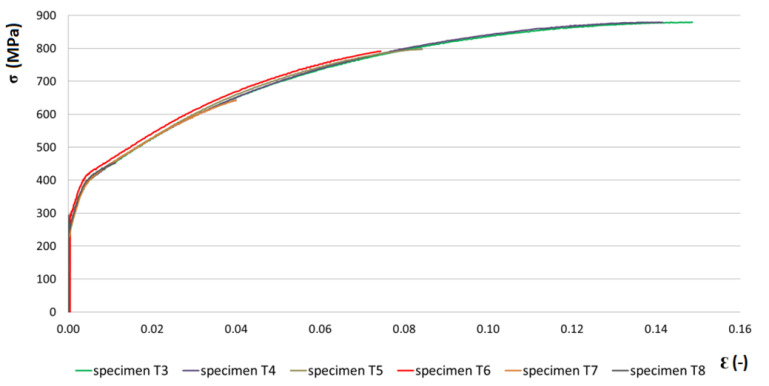
Stress–strain curves of TRIP steel for specimens T3–T8.

The results of the hydraulic bulge test, in which the stress–strain behavior was determined at individual degrees of plastic deformation (different bulge heights) are shown in Figure 6. From Figure 6 we can clearly state that the stress–strain behavior is almost identical in individual samples. It differs only in the amount of deformation. There are currently several models for determining the stress–strain dependence in the bulge test, which are based mainly on the change in the thickness of the material of the spherical dome during plastic deformation. The device we use allows us to determine the dependence of stress on deformation according to seven different models. For further experiments and simulations, the same model of determining the stress–strain dependence in the biaxial tensile test was used. The results of the bulge test were compared with the numerical results. The thickness values of the samples after the bulge test were compared and evaluated with numerical thickness values.

**Figure 7:**
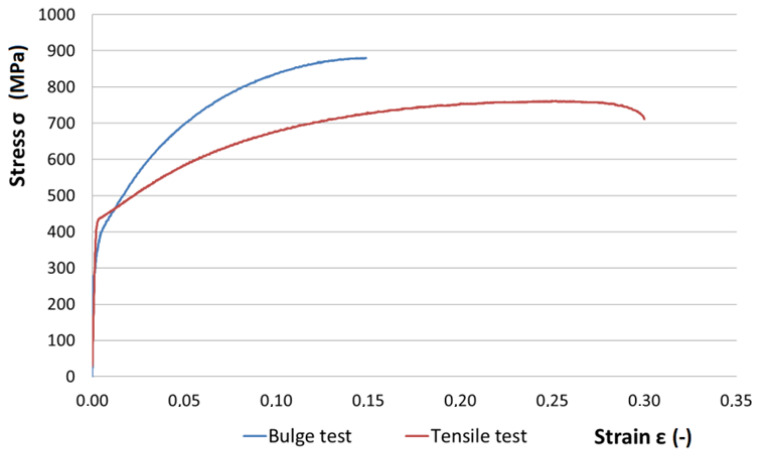
Stress–strain dependence from the uniaxial tensile test and the bulge test.

Figure 7 shows the stress–strain behavior comparison for the uniaxial tensile test and the bulge test. A much larger deformation was achieved using the uniaxial tensile test compared to the biaxial test. In the bulge test, the strain hardening of the material is higher than it is in the uniaxial tensile test.

**Figure 8:**
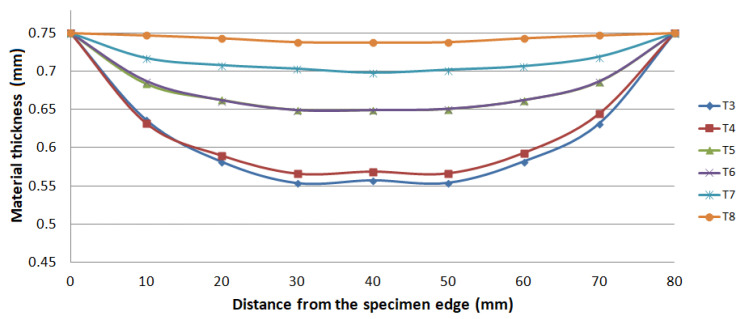
Material thickness values in 0° direction after bulge test.

**Figure 9:**
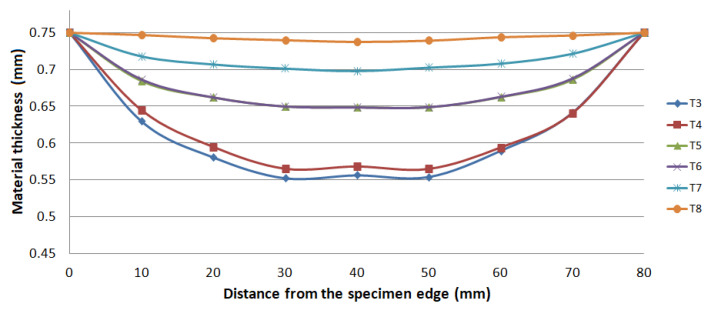
Material thickness values in 90° direction after bulge test.

**Figure 10:**
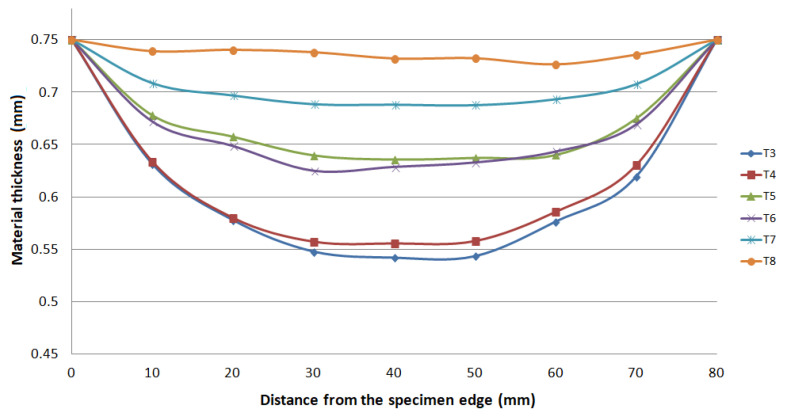
Material thickness values in 0° direction after bulge test evaluated by ARGUS system.

**Figure 11:**
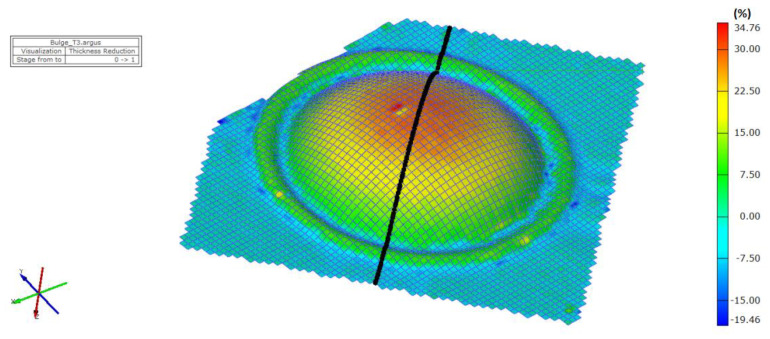
Material thinning after bulge test of T3 sample evaluated by ARGUS system.

**Figure 12:**
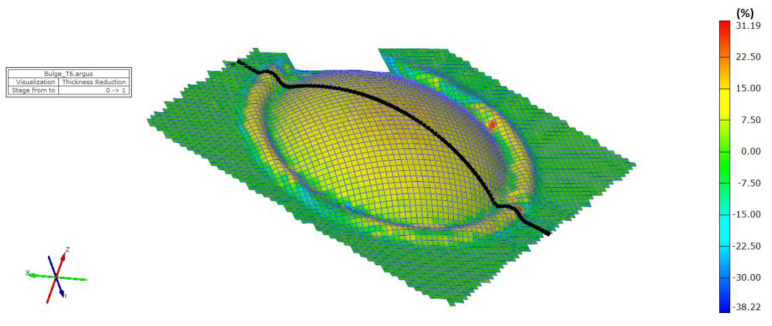
Material thinning after bulge test of T6 sample evaluated by ARGUS system.

The thickness results of the specimen are presented in Figure 8 and Figure 9 after bulge testing, which were measured using the digital point micrometer. The values calculated by ARGUS optical measuring system using GOM Inspect software are presented in Figure 10. Thinning maps of T3 and T6 samples are presented in Figure 11 and Figure 12. The estimated thicknesses using GOM Inspect software show some asymmetry. The asymmetry may be caused by various factors, such as the accuracy of the measuring equipment, insufficient quality of the tested surface, inappropriate quality of the etched deformation net, imperfect lighting conditions during scanning of the deformation net, and the grid points interpolation.

### 3.2. Simulation Bulge Test Results

**Figure 13:**
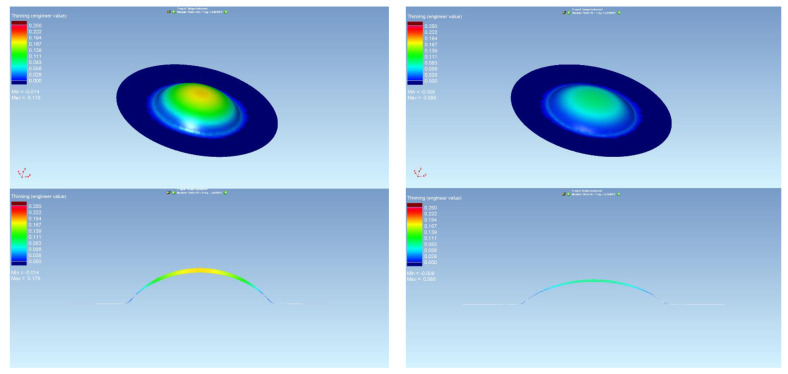
Comparison of predicted thinning values of samples T3 (**left**) and T6 (**right**) using software with explicit solver in program environment.

**Figure 14:**
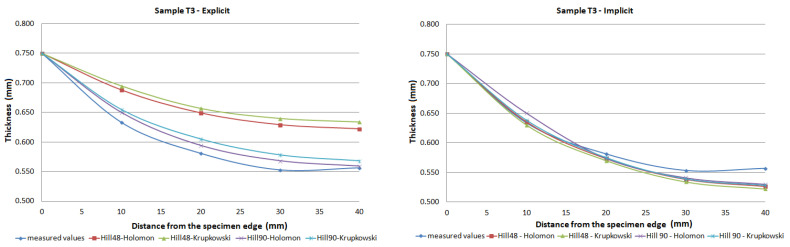
Comparison of predicted and measured thickness values of sample T3 using different types of yield criteria, hardening laws and integration scheme.

**Figure 15:**
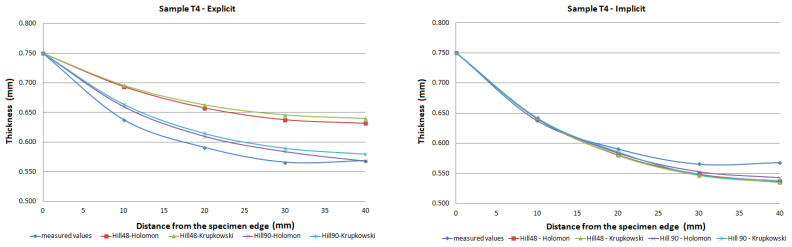
Comparison of predicted and measured thickness values of sample T4 using different types of yield criteria, hardening laws and integration scheme.

**Figure 16:**
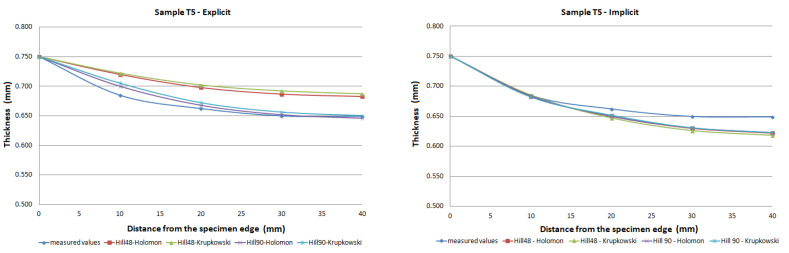
Comparison of predicted and measured thickness values of sample T5 using different types of yield criteria, hardening laws and integration scheme.

**Figure 17:**
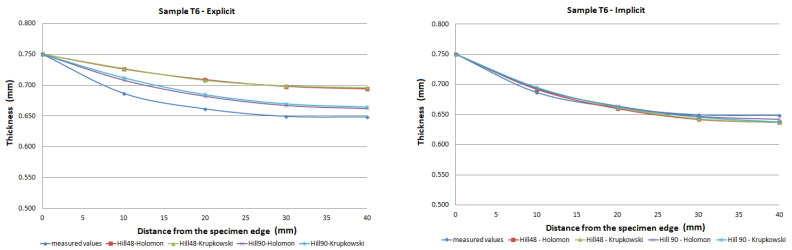
Comparison of predicted and measured thickness values of sample T6 using different types of yield criteria, hardening laws and integration scheme.

**Figure 18:**
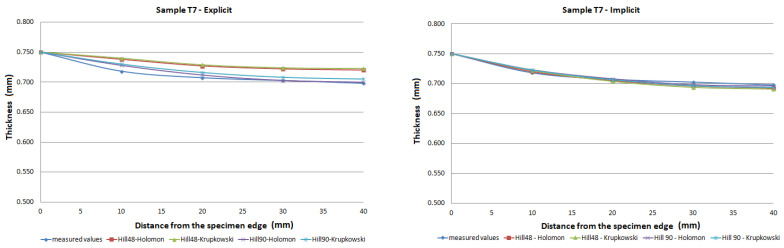
Comparison of predicted and measured thickness values of sample T7 using different types of yield criteria, hardening laws and integration scheme.

**Figure 19:**
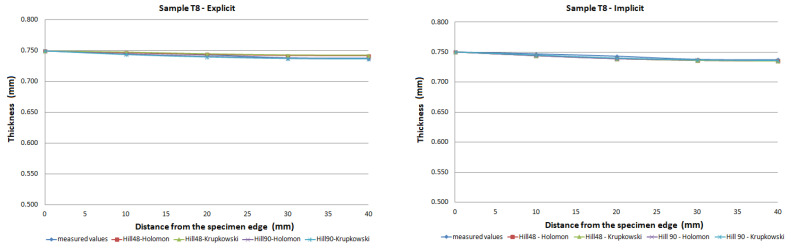
Comparison of predicted and measured thickness values of sample T8 using various yield criteria, hardening laws, and integration scheme.

The FE analysis aimed to analyze the thickness differences caused by changing hardening models and yield criteria in simulation. Additionally, the impact of the integration scheme (implicit/explicit) was evaluated. The comparison of predicted thickness values of samples T3 and T6 using software with explicit solver are presented in Figure 13. The simulation results are presented using graphs in Figure 14, Figure 15, Figure 16, Figure 17, Figure 18 and Figure 19.

The predicted thickness of the formed part showed the best correlation with measured thickness values when the Hill90 yield surface model combined with the Hollomon hardening model was used in simulation software with the explicit solver. The combination of the Hill90 yield surface model and Krupkowski hardening model showed the best correlation of thickness results when simulation software using implicit solver was used. Overall, smaller deviations of predicted thickness were observed for samples with lower deformation, lower dome height (samples T7, T8) and higher deviations of thickness were observed in samples with greater deformation, higher dome height (samples T3, T4). The results obtained using software with implicit solver showed a better correlation with experimental thickness results than results obtained using the software with explicit solver.

Mean deviations of predicted thickness *t* from the measured thickness values are shown in Table 5 and Table 6. Mean deviations were calculated according to the formula:

(7) $$Dev\;X=\frac{X_{sim}-X_{exp}}{X_{exp}}\cdot 100\;\left[\%\right]$$

where *Dev X* is a mean deviation of predicted thickness value from the experimental thickness value, *X_sim_* is the simulation thickness value and *X_exp_* is the experimental thickness value.

## 4. Discussion

Previously published research works regarding forming prediction of bulge test specimens focused predominantly on the analysis of material parameters and their effect on the forming prediction accuracy in numerical simulations [17,37,38,39]. The presented research focused on the thickness analysis of the bulge specimen made of TRIP steel. Its aim was to predict the bulge test by numerical simulation, where the impact of yield criteria and hardening models on the accuracy of the simulation was studied. The experimental results of sample thickness were obtained using two different methods: tactile and optical measurement. Optical measurement of thickness showed a good correlation with the conventional, tactile measurement. The numerical results showed that the hardening law has a substantial impact on the results of the bulge test simulation, and thickness prediction of the bulge test specimen. The hardening model describes the development of the plasticity surface during deformation/forming (changes in its size, shape, and/or its displacement) [4]. The calculations indicate a higher correlation of the Hollomon hardening law with the experimental results when the Hill48 yield criterion is also used in the simulation (Table 5 and Table 6). The same can be stated for the combination of the Hollomon hardening law with the Hill90 yield criterion, but only in software with the explicit solver. The results obtained with the use of the Krupkowski hardening law showed less accurate thickness results than the Hollomon law in most cases. Similar results regarding the impact of the hardening law on the accuracy of forming prediction of TRIP steel are stated by Mulidrán et al. [40].

Furthermore, the yield criteria influence on the thickness accuracy prediction was evaluated. Hill48 and Hill90 yield criteria were combined with isotropic hardening laws. The comparison of yield surface (Figure 5) shows that the yield stress for Hill90 is higher in the borderline cases of biaxial tension. Both yield surfaces reached the same values in uniaxial tension. When the bulge test is performed, the biaxial strain prevails. The higher yield strength value for the biaxial strain of the Hill90 yield surface model will also be reflected in the results of thickness prediction. This makes Hill90 more appropriate for the given type of strain, which is in order with the comparison of Hill48 and Hill90 for biaxial strain (Table 5 and Table 6). The impact of yield criterion on the predicted thickness results was evaluated by Tomas et al. [31], in their work box-shaped product made of DC06EK steel was studied. They found that the Hill48 yield criterion with isotropic hardening law was more accurate than Hill90 with isotropic hardening law. Numerical results of thickness showed a better correlation with experimental results when the Hill90 yield criterion was used in most cases. The impact of the solver type was also evaluated. The software with implicit solver showed a much better correlation with experimental thickness values (sample T3, mean deviation of 2.59 %) than software with explicit solver (sample T3, mean deviation of 12.97 %) when the Hill48 yield criterion was used with both hardening laws. Use of the Hill90 yield criterion showed more accurate results when implicit solver was utilized in comparison with explicit solver, in most cases.

The degree of deformation of a bulge test specimen, and its impact on the numerical prediction of thickness accuracy was studied. Based on the numerical results, it can be stated that the accuracy of thickness prediction for samples with greater deformation (sample T3, Figure 14) is less accurate in comparison with samples with smaller deformation (sample T8, Figure 19). This phenomenon can be attributed to the impact of the hardening law on the accuracy of the development of the plasticity surface during deformation.

## 5. Conclusions

The forming prediction accuracy of high-strength steel can pose a challenge in numerical simulation. This research investigated the influence of yield criteria and hardening laws on the accuracy of thickness prediction of bulge test specimens made of TRIP RAK 40/70 steel with the use of the FE method. Additionally, the impact of solver type and degree of deformation on the accuracy of numerical simulation was studied. The results lead to the following findings:

- Hollomon’s hardening law exhibited better accuracy in predicting thickness in comparison with the Krupkowski hardening law in most cases.
- The Hill90 yield criterion was found to be more suitable for simulation of bulge test using TRIP steel.
- Software with implicit solver and triangular shell elements showed better accuracy in predicting thickness of deformed sheet in bulge test than software with explicit solver and hexagonal shell elements in most cases.
- The degree of deformation impacted the simulation accuracy, while thickness prediction of samples with greater deformation was less accurate in comparison with samples with lower deformation and lower bulge height.

## Figures and Tables

**Table 1:** The chemical composition of the RAK 40/70 Z100MBO steel.

C (%)	Mn (%)	Si (%)	P (%)	S (%)	Al [%]	Nb (%)	Ti (%)	Mo (%)	Cr (%)
0.204	1.683	0.200	0.018	0.003	1.730	0.004	0.009	0.008	0.055

**Table 2:** The mechanical properties and formability parameters of the RAK 40/70 Z100MBO.

Dir. (°)	E (GPa)	R_p0.2_ (MPa)	R_m_ (MPa)	A_80_ (%)	*r* (-)	*r_m_* (-)	Δ*r* (-)	*n* (-)	*n_m_* (-)	Δ*n* (-)
0		441	766	27.9	0.680			0.293		
45	210	442	762	25.4	0.805	0.804	−0.003	0.294	0.290	−0.009
90		450	766	25.9	0.926			0.278		

Legend: E = Young’s modulus, R_p0.2_ = yield stress, R_m_ = ultimate tensile strength, A_80_ = total elongation, r = plastic strain ratio, n = strain-hardening exponent, n_m_ = average value of strain-hardening exponent, r_m_ = average value of plastic strain ratio Δr = planar anisotropy of plastic strain ratio, and Δn = planar anisotropy of strain-hardening exponent.

**Table 3:** The anisotropy parameters describing the yield criteria.

*r*_0_ (-)	*r*_45_ (-)	*r*_90_ (-)	*σ*_0_ (MPa)	*σ*_45_ (MPa)	*σ*_90_ (MPa)	*σ_biax_* (-)
0.680	0.805	0.926	441	442	450	1.01

**Table 4:** Hollomon and Krupkowski material model constants.

Model	*K* (MPa)	*n* (-)	*φ*_0_ (-)
Hollomon	1331	0.219	-
Krupkowski	1336	0.227	0.00765

**Table 5:** The mean deviations (%) of predicted thickness values from the experimental thickness values using software with the explicit solver.

Sample	Hill48 Hollomon	Hill48 Krupkowski	Hill90 Hollomon	Hill90 Krupkowski
**T3**	11.39	12.97	2.20	3.72
**T4**	11.00	11.94	2.84	3.85
**T5**	5.41	5.98	1.06	1.57
**T6**	6.81	6.90	2.83	3.32
**T7**	2.82	3.12	0.65	1.18
**T8**	0.24	0.38	0.19	0.28

**Table 6:** The mean deviations (%) of predicted thickness values from the experimental thickness values using software with the implicit solver.

Sample	Hill48 Hollomon	Hill48 Krupkowski	Hill90 Hollomon	Hill90 Krupkowski
**T3**	2.08	2.59	2.41	1.97
**T4**	2.40	2.60	1.90	2.18
**T5**	2.05	2.43	1.97	1.94
**T6**	0.88	0.90	0.84	0.79
**T7**	0.55	0.78	0.45	0.53
**T8**	0.42	0.42	0.37	0.29

## Data Availability

The data that support the findings of this study are available from the corresponding author upon reasonable request.
